# Role of physical activity and sedentary behavior in venous thromboembolism: a systematic review and dose-response meta-analysis

**DOI:** 10.1038/s41598-024-73616-0

**Published:** 2024-09-27

**Authors:** Gangpu Wang, Bo Han, Guofeng Dai, Ying Lian, Melanie L Hart, Bernd Rolauffs, Huanan Chen, Chengbin Tang, Chengqiang Wang

**Affiliations:** 1 Department of General Surgery, The Fourth People’s Hospital of Jinan City, Jinan, China; 2 G.E.R.N. Research Center for Tissue Replacement, Regeneration & Neogenesis, Department of Orthopedics and Trauma Surgery, Faculty of Medicine, Medical Center—Albert-Ludwigs- University of Freiburg, 79108 Freiburg in Breisgau, Germany; 3 Department of Medical Record Management and Statistics, The First Affiliated Hospital of Shandong First Medical University, Shandong Provincial Qianfoshan Hospital, Jinan, China; 4 Cardiovascular disease center, Northern Jiangsu people’s Hospital, Yangzhou, China; 5 Department of Cardiac Surgery, The Affiliated Yantai Yuhuangding Hospital of Qingdao University, Yantai, China

**Keywords:** Physical activity, Sedentary behavior, Venous thromboembolism, Dose-response meta-analysis, Diseases, Health care, Risk factors

## Abstract

Increasing studies have investigated the link between physical activity (PA) and sedentary behavior with venous thromboembolism (VTE) but the existing findings are not consistent and the independent relationship is uncertain. This meta-analysis aimed to comprehensively assess the shape of dose-response relationship between PA and sedentary behavior with VTE and further explore whether the relationship is independent after mutual adjustment. We systematically searched PubMed, Embase and Web of Science from inception to August 1, 2024. PA exposures were converted into MET-h/wk. Categorical meta-analyses and a cubic spline model were performed to evaluate the association between PA, sedentary behavior and VTE. Twenty-five articles including 31 studies were included. A curvilinear dose-response relationship between PA and VTE was observed, with steeper gradients even at lower PA levels. After adjustment for sedentary behavior, higher level of PA was independently associated with a reduced VTE risk (OR = 0.83, 95%CI:0.77–0.89). Based on population attributable fraction analyses, 2.37% (95%CI: 1.90-2.85%) of incident VTE could have been prevented if all adults had achieved half the PA minimum recommended level. A linear dose-response relationship between sedentary behavior and VTE risk was found, and there was a 2% higher risk of VTE (OR = 1.02, 95%CI: 1.00-1.03) for 1 h increment of sedentary behavior per day. After adjustment for PA, sedentary behavior was independently associated with an increased VTE risk (OR = 1.19, 95CI%:1.01–1.39). Our analyses demonstrated PA and sedentary time were indeed independently associated with the risk of VTE after mutually adjusting for sedentary time or PA, highlighting a unique perspective on their individual contributions. Further studies assessing the effects of different combinations of PA and sedentary time for assessing joint effects on VTE are needed.

**Supplementary Information:**

The online version contains supplementary material available at 10.1038/s41598-024-73616-0.

## Introduction

Venous thromboembolism (VTE) is a serious condition characterized by blood clots forming in the veins, including pulmonary embolism (PE) and deep vein thrombosis (DVT). As one of the five most prevalent vascular conditions worldwide, VTE is a significant public health concern. It can severely impact daily life, leading to pain, swelling, reduced mobility, and a heightened risk of recurrent thrombotic events. Among adults the lifetime risk of VTE is approximate 8%, and there are nearly 10 million VTE events every year worldwide^1,2^. It is reported that about 20% of individuals with VTE events die within 1 year^3^, and VTE is widely regarded to be a substantial contributor to the global burden^4,5^. Accumulating evidence indicated that VTE is a multifactorial disease^6,7^ and can be potentially preventable through effective strategies.

Among the modifiable factors, physical activity (PA) is one of the main lifestyle-related factors for VTE that individuals are exposed to daily. Accumulating studies focus on the potential role of PA in the development of VTE, however, the benefit of PA for VTE is an ongoing debate. One meta-analysis of prospective studies has suggested that, compared with people with the lower levels of PA, those with higher PA levels had 13% (95% CI 5%-21%) lower odds of developing VTE^8^. However, another review without meta-analysis provided diverging results as to the association between high levels of PA and risk of VTE. This review indicated that while several studies have shown an association between high PA levels and an increased risk of VTE, not all studies reached the same conclusion. Therefore, more research using appropriate methodologies is needed to further explore the relationship between PA and VTE risk^9^. Furthermore, several additional studies have since been published on the association^10–12^ but the findings have been mixed in the absence of an update and a comprehensive meta-analysis. Accurate estimates of a dose-response association between PA and VTE are important and may be not only useful to promote awareness of PA in prevention of VTE but also could be helpful in guiding decisions at the policy level. Nevertheless, existing reviews estimated PA exposure by using binary categorization^9^, but this approach results in loss of information on PA doses and does not provide the variation in VTE risk across a range of PA doses.

Sedentary behavior is highly prevalent in our daily lives^13^, which is related but distinct from physical inactivity^14^, considering that someone can meet the PA recommendations despite being highly sedentary throughout the remaining waking hours. Some previous studies found that the associations between sedentary behavior and clinical health outcomes are independent of PA^15–17^. In line with this, it has been shown that prolonged sitting may cause vein compression, thereby reducing blood flow and increasing the risk of VTE^18^. Importantly, the association between sedentary behavior and VTE have not yet been systematically investigated, and it remains unclear whether PA attenuates the potential association of sedentary behavior with VTE. Whereas there are international public health PA recommendations^19^, there was insufficient evidence to quantify a sedentary behavior threshold^20^. Thus, quantitatively synthesizing evidence on sedentary behavior and VTE is required to inform public health officials in creating guidelines.

To bridge the knowledge gap, we conducted an updated and comprehensive systematic review and meta-analysis to investigate the shape of dose-response associations between PA and sedentary behavior with VTE, and whether the relationships of these two exposures with VTE are independent after mutual adjustment. We also further assessed the population attributable fraction (PAF) to estimate potential population changes in VTE that may be prevented by higher PA levels.

## Methods

This systematic review was conducted following the guidelines of the Preferred Reporting Items for Systematic Reviews and Meta-Analyses (PRISMA)^21^, The PRISMA checklist is exhibited in **Supplementary Table 1.** This study was prospectively registered in PROSPERO with the PROSPERO number being CRD42023424994.

### Search strategy

A comprehensive search was conducted in electronic databases including PubMed, Web of Science and Embase from inception to August 1, 2024. A list of search terms based on [Title/Abstract] was as follows: (Exercise OR physical *activit* OR sedentary behavior OR recreational activity OR sports OR active transport OR active transportation OR household activity OR housework OR non-exercise activity OR activities of daily living OR physical fitness OR leisure activities) and (Venous Thromboembolic OR deep vein thrombose OR pulmonary embolism OR vein thrombosis OR thrombosis OR thromboembolism OR lung embolism). Besides, we also searched manually the references list of relevant articles and reviews.

### Study selection

The following criteria were used to determine if a study was eligible for inclusion: (1) the study design is a cohort, case control or cross-sectional study to assess the relationship between PA or sedentary behavior and VTE; (2) reported effect estimates as hazard ratios (HRs), odds ratios (ORs), or relative risks (RRs) with 95% confidence intervals (CIs); (3) the exposures of interests were physical activity or sedentary behavior. If the data of multiple articles came from the same study, we chose the articles which reported the most informative PA level or had the largest sample size. Two authors independently selected eligible articles based on the title, abstract and full-text assessment. A third author resolved any disagreements.

### Data extraction and quality assessment

The following information was extracted including: first author, publication year, study name, study design, location, characteristics of participants, sample size, exposure levels, case number per level of PA and sedentary behavior, total persons or person-years per PA and sedentary behavior level, the duration of follow up, the ORs/RRs/HRs for VTE with 95%CIs for each PA and sedentary behavior category, and adjusted variables in original analyses. The quality assessment of cohort and case-control study were evaluated using the Newcastle –Ottawa (NOS) scale^22^. Data extraction and quality assessment were independently conducted by the authors (HN. C. and YL).

### PA exposure harmonization

A comprehensive PA exposure harmonization was conducted using a common metric in metabolic equivalent task hours per week (MET-h/wk), which could integrate different intensity and duration of PA in a week. The mean intensity of light PA, moderate PA, moderate to vigorous PA and vigorous PA were defined as 3, 4, 4.5 and 8 METs, respectively^23^. When MET was not provided directly in original articles, PA volume (MET-h/wk) was calculated by multiplying the duration (median or midpoint) of the reported category by its assigned MET value^24^. If the lowest category of duration or frequency was open-ended, the lower boundary was considered as 0. If the highest category of duration was not reported, the width of the interval was thought to be equal to its neighboring category. If PA frequency per week was only provided, PA frequency per week was converted to h/week by assigning a dose of 45 min per session^25^. If the duration was not provided, the duration of per session was assumed as 45 min. If the intensity of PA was not provided in the articles, we assigned the intensity as 4.5 METs^25^. The dose assignment calculations are shown in **Supplementary Table 2**.

### Statistical analysis

All statistical analyses were executed by STATA version 12 (Stata Corp, College Station, Texas, USA). For categorical meta-analysis, the ORs and 95% CIs were pooled for highest vs. lowest category of PA and sedentary behavior. Cochran *Q* test and *I*^*2*^ were used to test the heterogeneity. *P* < 0.1 was considered as statistical heterogeneity for *Q* statistics. The value of *I*^2^ ranging from 0 to 25% was considered as no heterogeneity, *I*^2^ < 50%, 50-75% and > 75% were considered as low, moderate and high heterogeneity, respectively. Fixed effects model was used if *I*^2^ < 50% or else the random effects model was employed. Subgroup analyses were performed to estimate the effect across potential factors including sex, age, location, study type, the number of cases, quality score, adjusted for sedentary behavior/PA and adjusted for BMI. Sensitivity analyses were adopted to assess the stability of results by excluding one study at a time. Potential publication bias was assessed by Begg and Mazumdar’s test, Egger’s test and funnel plots. We further conducted trim-and-fill approach to explore the adjusted effect size when there was publication bias.

Studies with at least three exposure categories were included in dose-response analyses. Dose-response relationships were estimated by using the Greenland and Longnecker^26^ and Orsini^27^ methods. Studies with at least three exposure categories were included in the analyses. Potential nonlinear relationships between MET-h/wk or sedentary behavior and risk of VTE were evaluated using restricted cubic splines (RCS), with 3 knots located at the 10th, 50th and 90th of the distribution of MET-h/wk or sedentary behavior. The *P* value for nonlinearity was calculated by testing the null hypothesis that the coefficient of the second spline was equal to zero. Additionally, RR per 10 MET-h/wk increment and per hour of sedentary time increment were calculated using two-stage generalized least squares regression, respectively.

PAF was calculated based on PA exposure levels in population. The PAF was calculated according to Levin’s formula^28^, and the RR estimates and the prevalence (P) of exposure were required in the formula^29^.

$$\:PAF=\frac{\text{P}(\text{R}\text{R}-1)}{\text{P}\left(\text{R}\text{R}-1\right)+1}$$

PAFs were calculated for three WHO recommended levels of PA^20^, including 5.25 MET-h/wk (which was approximate half the minimum recommended level, 5.625 MET-h/wk), 12 MET-h/wk (which approached the minimally recommended level of 11.25 MET-h/wk), and 22.5 MET-h/wk (the upper bound of recommended levels). The RR is the pooled RR of VTE in dose-response curves associated with PA.

### Patient and public involvement

There was no direct patient or public involvement in this review.

## Results

### Study characteristics

**Fig. 1:**
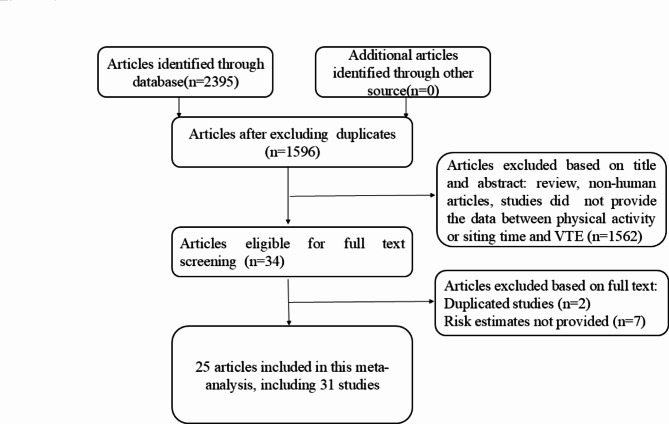
Results of the study selection process.

The selection procedure is shown in Fig. 1. Twenty-five articles including 31 studies with 1,787,740 individuals and 37,694 incident cases of VTE were included in present study, including 23 cohort and 8 case-control studies^11,12,18,30–51^. Among the included articles, 21 articles provided the information on PA and VTE, and 5 articles provided sedentary behavior and VTE. The PA/sedentary behavior level in all included studies were assessed by self-reporting. 6 articles were based in America, 17 articles in Europe, 1 article in middle East countries, and 1 article in both America and Europe. The mean quality scores were 7.1. The study characteristics are shown in Table 1.

**Table 1:** Descriptive information of included studies.

Authors	Study name	Location	Females (%)	Baseline age	Baseline BMI	Study design	Samples/cases	Exposure	Adjusted variables	Quality score
Armstrong et al. ^41^	Million Women	UK	100	55.9	26	cohort	999,708/ 12,859	Any activity	BMI, age, smoking, alcohol, socioeconomic status and region	7
Lindqvist et al. ^31^	MISS	Sweden	100	25–64	NA	cohort	24,098/ 219	Regular exercise	age, cancer, parity, BMI, combined oral contraceptives, cigarettes, alcohol consumption	9
Stralen et al. ^33^	Cardiovascular Health	US	55.26	≥ 65	NA	cohort	5534/ 171	Walking, jogging	sex, age, race, BMI, self-reported health	8
Nymberg et al. ^37^	WHILA	Sweden	100	50–64	NA	cohort	6916/ 220	PA	age, smoking, former smoking, waist circumference, varicose veins and hypertension	6
Bergendal et al.^30^	TEHS	Sweden	100	37.93	NA	case-control	1441/ 767	Exercise	age, BMI, smoking, use of hormones, bedrest/minor trauma, surgery and cast, the prothrombin, mutation and/or factor V Leiden	8
	TEHS	Sweden	100	58.24	NA	case-control	1100/ 524	Exercise	age, BMI, smoking, use of hormones, bedrest/minor trauma, surgery and cast, the prothrombin, mutation and/or factor V Leiden	8
Evensen et al. ^34^	Tromsø Study	Norway	52.4	47	25.3	cohort	30,002/ 531	PA	age, sex, BMI, history of cardiovascular disease and cancer	6
Johansson et al ^42^	VEINS	Sweden	51	46.3	25.84	cohort	92,911/ 1645	PA	age, BMI, hypertension, smoking, educational level cancer	8
Yuan, et al ^12^	COSM	Sweden	0	60.3	25.8	cohort	36,193/ 2043	PA	age at baseline, BMI, education, energy intake, history of diabetes and fracture, aspirin use, cancer, smoking, alcohol and coffee consumption, modified DASH diet score	8
	SMC	Sweden	100	61.5	25	cohort	30,137/ 1784	PA	age at baseline, BMI, education, energy intake, history of diabetes and fracture, aspirin use, hormone therapy, cancer, smoking, alcohol and coffee consumption, DASH score	8
Kunutsor et al ^38^	KIHD	Finland	0	53	26.9	cohort	2259/ 145	Skiing	age, BMI, SBP, prevalent coronary heart disease, smoking status, history of T2DM, total cholesterol	8
Kim et al.^36^	NHS	US	100	30–55	26.84	case-control	78,936/ 889	Sitting time, PA	BMI	7
	NHS II	US	100	25–42	27.49	case-control	1766/ 447	Sitting time, PA	BMI	7
	HPFS	US	0	40–75	26.47	case-control	1808/ 798	Sitting time, PA	BMI	7
Lutsey et al. 43	IWHS	US	100	65.9	NA	cohort	40,377/ 2137	PA	age, education, smoking status, BMI	6
MacDonald et al ^11^	E3N study	French	100	51.1	22.9	cohort	91,707/ 1649	PA	age, statin use, education, parity, menopausal status, MHT use, type of menopause, BMI	6
Wattanakit et al ^35^	ARIC	US	55	54.1	NA	cohort	15,340/ 468	PA	age, race, center, gender and BMI	9
Holst et al ^44^	CCHT	Denmark	100	51	24.97	cohort	9712/ 510	Leisure time PA	age and calendar time.	6
	CCHT	Denmark	0	51	24.97	cohort	8430/ 432	Leisure time PA	age and calendar time.	6
Olson et al ^32^	REGARDS	US	55.31	65.04	29.31	cohort	27,082/ 263	Leisure time PA	age, gender, income, education, race, region, interaction.	7
Solli et al ^39^	DNPR	Danish	53.1	25–79	28.2	cohort	57,523/ 508	PA	no	5
Stralen et al ^45^	MEGA	Netherlands	53.8	47.06	26.19	case-control	7860/ 3608	PA	sex, age, and BMI	7
Nymberg et al. ^40^	MPP	Sweden	0	43.57	24.65	cohort	22,444/ 1635	Exercise	no	6
	MPP	Sweden	100	49.58	24.32	cohort	10,902/ 977	Exercise	no	6
Engbers et al ^48^	AT-AGE	US and Netherlands	51.51	≥ 70	NA	case-control	832/ 401	Sleeping/sitting time	age, sex, study center, and comorbidities.	8
Kubota et al ^47^	ARIC	US	54.84	54.12	27.65	cohort	15,158/ 691	Watch TV time	age, gender, race, center, smoking status, PA, eGFR, cardiovascular disease and BMI.	9
Johannesen et al. 48	CCHS and CGPS	Danish	56.67	20–64	NA	cohort	78,936/ 911	Sitting time	gender, age, smoking, education, use of hormones, BMI, cancer, cardiovascular disease, births, and major surgery	8
West et al. ^46^	NA	UK	36.45	48.82	NA	case-control	203/ 97	Sitting time	seated immobility at work, maximum hours seated, maximum hours seated without getting up	6
Michael Shapiro et al ^49^	IDF	Israeli	100	18.8	22.35	cohort	160,718/ 52	Strenuous PA	Age, BMI, Health status, Contraceptive use	5
Nzechukwu M Isiozor et al ^50^	KIHD	Finnish	0	52.3	26.7	cohort	1899/ 127	Physical score	Age, alcohol consumption, socioeconomic status and family history of coronary heart diseas	6
P Wändell et al ^51^	ULSAM	Sweden	0	50	24.9	cohort	2294/ 186	Hard PA	LDL, HDL, SBP, BMI, diabetes, and smoking	7

Abbreviations listed in alphabetical order: MISS: Melanoma Inquiry of Southern Sweden study. WHILA: Women’s Health In the Lund Area study. TEHS: Thrombo Embolism Hormone Study. VEINS: Venous Thromboembolism in Northern Sweden. COSM: Cohort of Swedish Men study. SMC: Swedish Mammography Cohort study. KIHD: Kuopio Ischemic Heart Disease Risk Factor study. NHS: nurses’ health study. HPFS: Health Professionals Follow-up study. IWHS: Iowa Women’s Health Study. E3N: Etude Epidémiologique auprès de femmes dela Mutuelle Générale de l’Education Nationale. ARIC: Atherosclerosis Risk in Communities study. CCHT: the Copenhagen City Heart Study. REGARDS: Reasons for Geographic and Racial Differences in Stroke Study. DNRP: Danish National Health Service study. MEGA: Multiple Environmental and Genetic Assessment study. MPP: Malmo Predictive Program study. AT-AGE: the Age and Thrombosis-Acquired and Genetic risk factors in the Elderly. ARIC: Atherosclerosis Risk in Communities study. CCHS: Copenhagen City Heart Study. CGPS: Copenhagen General Population Study. BMI: body mass index. eGFR: Estimated glomerular filtration rate.

### PA and VTE

**Fig. 2:**
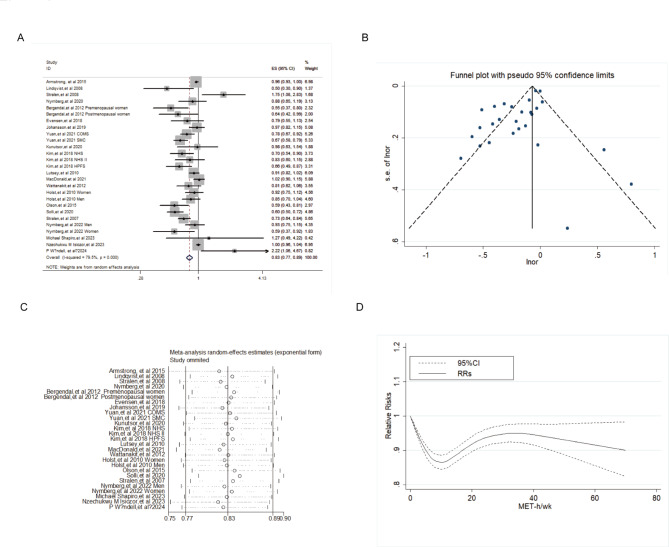
**A**: Forest plot of the pooled effect on VTE for the highest category of PA versus lowest category. **B**: Funnel plot. **C**: Sensitivity analysis. **D**: Dose-response relationship between PA and VTE.

Twenty-one articles including 27 studies with 35,594 VTE cases among 1,692,611 participants were included to evaluate the relationship between PA and VTE. The pooled OR for VTE in highest PA level was 0.83 (95%CI 0.77–0.89), with high heterogeneity (*I*^*2*^ = 79.5%, *P* < 0.01, shown in Fig. 2A). Although the Begg and Mazumdar’s test did not signal publication bias (Kendall’s tau = 0.11, *P* = 0.39), the Egger’s test (*P* = 0.04) suggested existence of publication biases (Fig. 2B). There was no study imputed using the trim-and-fill method, indicating that there was no publication bias and the finding was steady.

In sensitivity analyses, by eliminating any study each time, the pooled risk did not substantially change, with the summary ORs ranging from 0.81 (95%CI: 0.75–0.89) to 0.84 (95%CI:0.79–0.90), confirming the stability of presenting results (Fig. 2C).

The estimated OR was 0.83 (95%CI:0.76–0.91) with BMI adjustment. After adjustment for sedentary behavior, PA was independently associated with a reduced VTE risk (OR = 0.72, 95%CI:0.61–0.85). More details were showed in Table 2.

### Sedentary behavior and VTE

**Fig. 3:**
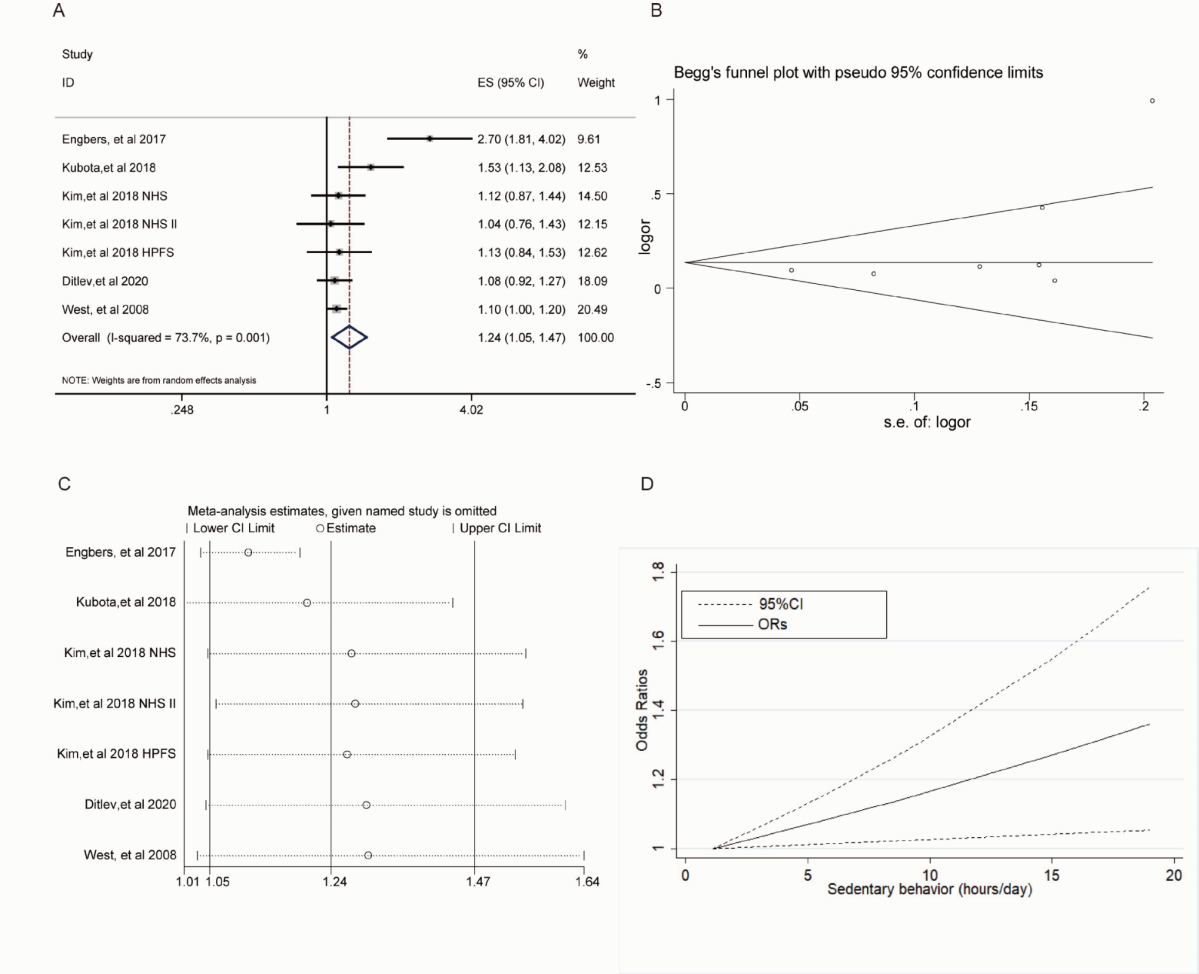
A: Forest plot of the pooled effect on VTE for the highest category of sedentary behavior versus lowest category. B: Funnel plot. C: Sensitivity analysis. D: Dose-response relationship between sedentary behavior and VTE.

Five articles including 7 studies with 101,153 participants and 4234 VTE events were included regarding relationship between sedentary behavior and VTE. Prolonged sedentary behavior was associated with an increased VTE risk (OR = 1.24, 95%CI: 1.05–1.47), with high heterogeneity (*I*^*2*^ = 73.7%, *P* < 0.01) (Fig. 3A**)**. Inspection of funnel plot (Fig. 3B) suggested existence of publication bias, while there was no indication in the Begg and Mazumdar’s test (Kendall’s tau = 0.43, *P* = 0.22) and Egger’s test (*P* = 0.17). Sensitivity analyses did not show substantive change from the pooled risk (Fig. 3C).

Sedentary behavior was associated with higher risk of VTE (OR = 1.14, 95%CI:1.02–1.28) after BMI adjustment. After adjustment for PA, sedentary behavior was independently associated with an increased risk of VTE (OR = 1.19, 95CI%:1.01–1.39). More details are shown in Table 2.

**Table 2:** Subgroup analyses on VTE risk with PA and sedentary behavior.

Characteristics	PA				Sedentary behavior			
	*n*	OR (95%CI)	I^2^	*P*	*n*	OR (95%CI)	I^2^	*P*
**All studies**	**27**	**0.83(0.77–0.89)**	**79.5**	**< 0.01**	**7**	**1.24(1.05–1.47)**	**73.7**	**< 0.01**
Sex								
M/F	7	0.80(0.66–0.97)	79.3	< 0.01	4	1.39(1.06–1.82)	86.6	< 0.01
F	13	0.81(0.73–0.90)	74.5	< 0.01	2	1.09(0.89–1.32)	0	0.72
M	7	0.89(0.77–1.03)	73.9	< 0.01	1	1.13(0.83–1.53)	-	-
Age								
<60 years	22	0.84(0.78–0.90)	76.3	< 0.01	6	1.15(1.04–1.20)	73.7	0.03
≥60 years	5	0.82(0.66–1.01)	83.4	< 0.01	1	2.70(1.81–4.02)	--	--
Location								
European	19	0.83(0.77–0.90)	81.90	< 0.01	0	--	--	--
America	7	0.81(0.68–0.97)	70.80	< 0.01	7	1.24(1.05–1.47)	73.7	< 0.01
Other	1	1.27(0.49–4.22)	0	0				
Study type								
Cohort	21	0.86(0.80–0.93)	78.3	< 0.01	4	1.16(0.99–1.35)	30.8	0.23
Case-control	6	0.71(0.64–0.78)	0	0.67	3	1.43(0.95–2.14)	89.2	< 0.01
Cases								
<500	11	0.90(0.77–1.06)	69.2	< 0.01	3	1.41(0.89–2.25)	89.4	< 0.01
≥ 500	16	0.79(0.72–0.87)	81.9	< 0.01	4	1.16(1.01–1.34)	25.1	0.26
Quality Score								
≤ 7	18	0.84(0.78–0.91)	78.8	< 0.01	4	1.10(1.02–1.19)	0	0.98
> 7	9	0.79(0.67–0.94)	71.9	< 0.01	3	1.60(0.97–2.63)	89.5	< 0.01
Adjusted for sedentary behavior/PA								
No	24	0.84(0.78–0.90)	80.2	< 0.01	3	1.36(0.99–1.87)	89.4	< 0.01
Yes	3	0.72(0.61–0.85)	0	0.57	4	1.19(1.01–1.39)	17.5	0.30
Adjusted for BMI								
No	8	0.80(0.68–0.95)	84.4	< 0.01	2	1.69(0.70–4.06)	94.6	< 0.01
Yes	19	0.83(0.76–0.91)	77.0	< 0.01	5	1.14(1.02–1.28)	8	0.36
BMI								
<26 kg/m^2^	17	0.81(0.65-1.00)	77.6	< 0.01	2	1.12(0.93–1.36)	0	0.97
≥ 26 kg/m^2^	4	0.65(0.53–0.79)	37.1	0.20	2	1.26(0.87–1.85)	66.3	0.09

### Dose-response relationship between PA, sedentary behavior and VTE

Eleven cohort studies with 22,120 VTE events were included in the dose-response analysis between PA and VTE, which showed a nonlinear dose-response relationship between PA and VTE (Fig. 2D, *P*_non−linear_<0.01), with steeper association gradients at lower PA volumes. There was the strongest protection against VTE when PA accumulated to 12 MET-h/wk in which, relative to adults not reporting any PA, those accumulating the recommended volume (12 MET-h/wk) had a 13% lower risk of VTE (RR = 0.87, 95%CI:0.84–0.90).

Four studies including 2,535 VTE cases were included restricted cubic spline analysis regarding between sedentary behavior and VTE. As shown in Fig. 3D, a linear dose-response association was present between sedentary behavior and VTE risk and there was a 2% higher risk of VTE for each 1 h increment of sedentary time per day (OR = 1.02, 95%CI: 1.00-1.03).

### Potential population impact

Based on PAF analyses, 2.37% (95%CI: 1.90-2.85%) of VTE could be prevented if all adults achieved half the minimally recommended level of PA (5.25 MET-h/wk). Moreover, 2.85% (95%CI: 2.13-3.60%) of VTE could be prevented if all adults reached 12 MET-h/wk of PA, which approached the WHO minimally recommended level of 11.25 MET-h/wk. (Table 3).

**Table 3:** Relative risk and potential PAF of VTE at three PA levels.

PA levels	RR	95%CI		PAF (%)	95%CI
5.25 MET-h/wk	0.89	0.87–0.91		2.37	1.90–2.85
12 MET-h/wk	0.87	0.84–0.90		2.85	2.13–3.60
22.5 MET-h/wk	0.94	0.91–0.96		1.23	0.81–1.90

## Discussion

This systematic review showed a reduced risk of VTE with higher levels of PA and an increased risk of VTE with higher levels of sedentary behavior. A nonlinear dose-response relationship was found between PA and VTE, with a steeper risk reduction of VTE even at levels below the recommendations from WHO. Moreover, the public health impacts of VTE risk associated with changing the population-level PA exposure quantifiably showed a linear dose-response relationship between sedentary behavior and VTE, with a 2% higher risk of VTE with every 1 h increment of sedentary behavior per day. Furthermore, we observed PA and sedentary time were indeed independently associated with the risk of VTE even after mutual adjustment for sedentary time or PA.

Our finding was consistent with a previous meta-analysis study demonstrating that, compared with inactive individuals, there was a 17% reduced risk of VTE with higher PA levels^8^. In our study, subgroup analyses showed that the VTE risk was attenuated after BMI adjustment but remained significant, and the effect of PA was somewhat stronger in those with higher BMI. Moreover, the protective effect of PA tended to persist after adjusting for sedentary behavior.

Estimating the dose-response relationship using a harmonized approach can address limitations of the existing evidence that lacks specific doses of PA on VTE. Using a harmonized approach, we showed for the first time that there was a nonlinear dose-response relationship in the association between PA and VTE, in which a steeper risk reduction of VTE was found even at levels below the recommendations from WHO. Of note, if population levels of PA achieved half the recommended volume equal to 75 min/week of moderate-to-vigorous PA (5.25 MET-h/wk), then 2.37% of VTE incidents would potentially have been prevented. Collectively, the observed relationships demonstrate potential benefits for initially starting with small amounts of PA and gradually increasing over time, which could provide further insights into the development of public health interventions.

Regarding sedentary behavior, the present study supported and extended existing evidence demonstrating a positive association between sedentary behavior and VTE. However, our study extended these studies and further evaluated the shape of the relationship quantitatively, which provided insights and associations with greater precision. Patterns of the relationship suggested a linear dose-response with a 2% higher risk of VTE with every 1 h increment of sedentary behavior per day. This is in line with a previous study showing that sedentary behavior increased the mortality of cardiovascular disease independent of PA^52^. Similarly, the pooled estimated effect from four independent studies on sedentary time after adjustment for PA indeed suggested the independent detrimental effect of sedentary behavior on VTE, providing further perspectives into the interaction between sedentary behavior and PA on the development of VTE^53^. The present finding on VTE are further in line with the guidelines issued by the WHO, stating that adults should limit the time of sedentary behavior and replace with PA of any intensity. In addition, previous subgroup analyses stratified by levels of PA indicated that longer sedentary time had particularly adverse effects in populations with lower levels of physical activity but the effects were eliminated with at least 1 h of moderate intensity PA per day^52^. And there were also some previous studies focused on the divergence association between the specific domains of sedentary behavior and health outcomes^54,55^. We were unable to stratify subgroups according due to the limited available data leading to uncertainty regarding joint effects for combinations of PA with sedentary behavior. A future study is required to assess the interaction between PA and sedentary behavior on the risk VTE.

Although the mechanisms cannot explain the observed associations, numerous physiological mechanisms support the potential effect of PA and sedentary behavior in VTE incidence. PA could improve circulation of blood vessels and lower the odds of blood stagnation in the lower extremities, thereby reducing the risk of VTE^56^. In addition, PA could confer protection via anti- inflammatory mechanisms since a physically active lifestyle is known to interrupt inflammatory processes and generally lower the risk of an elevated inflammatory status^57^. On the other hand, sitting time can increase the levels of circulating inflammatory factors and hemostatic parameters, which increase VTE risk, especially deep vein thrombosis^58^. Moreover, the prolonged sitting time can inhibit the venous blood returning from the lower limbs and further promote venous stasis^59,60^. Sitting for a long time can weaken the muscle contraction, affecting the metabolism and blood vessel health, all of which lead to the development of VTE.

The present study has several strengths. This is the first dose-response meta-analysis that includes more original studies with a larger sample size to qualify the relationship using a novel harmonized exposure measurement, which reported how the risk of VTE varies along with the PA exposure used as a continuous variable. Second, sensitivity analyses further indicated the stability of the present findings. And detailed subgroup analyses were separately conducted and stratified with and without some adjusted potential confounders including BMI, PA for sedentary behavior, sedentary behavior for PA, thereby providing valuable insights for prevention strategies considering these variables. There are also some limitations that should be noted. Firstly, the assessment of PA and sedentary behavior in most included studies relied on single-time, self-reported measurements, which may lead to inaccurate association estimates. Future studies utilizing objective devices that measure PA and capturing trajectories of exposure are required to examine the associations. Secondly, almost all of the included studies are observational designed rather than interventional studies, which does not allow for inferences about causality. Thirdly, there were insufficient studies in dose-response analysis and subgroup analysis to distinguish the effect of different types of sedentary behavior with diverse domains of PA on VTE.

## Conclusion

The present systematic review found that PA and sedentary time were indeed independently associated with the risk of VTE after mutually adjusting for sedentary time or PA. The dose-response relationship curves suggested a sharp risk reduction of VTE even below the recommended levels of PA, and potentially a slight risk increase beyond a higher PA threshold, suggested by the restricted cubic splines (RCS) model. There was a 2% higher VTE risk for 1 h increments in sedentary behavior per day. The findings therefore have substantial implications, which could inform national policy and intervention strategies in optimizing and promoting the benefits of promoting PA as well as synergistically reducing sedentary behaviors. Further cohort studies and randomized controlled trials assessing the effects of different combinations and types of PA and sedentary times using objective devices for assessing joint effect of PA and sedentary time on VTE are needed.

**Supplemental information**.

Supplemental information can be found in the attachment.

## Electronic supplementary material

Below is the link to the electronic supplementary material.

Supplementary Material 1

Supplementary Material 2

## Data Availability

All data are available in the main text or as a supplementary file. Additional requests can be made to the corresponding author upon reasonable request.
