# Supplementary material for: Role of physical activity and sedentary behavior in venous thromboembolism: a systematic review and dose-response meta-analysis
Source: Sci Rep. 2024 Sep 27;14:22088. doi: 10.1038/s41598-024-73616-0 (PMC11437044; doi:10.1038/s41598-024-73616-0)
Supplement: Supplementary file 1 — Supplementary Material 1 [file 41598_2024_73616_MOESM1_ESM.docx]

**Supplementary Table 1** | PRISMA Checklist.

| Section and  topic | Item  # | Checklist item | Reported on page No |
| --- | --- | --- | --- |
| TITLE | | | |
| Title | 1 | Identify the report as a systematic review, meta-analysis, or both | 1 |
| ABSTRACT | | | |
| Structured summary | 2 | Provide a structured summary including, as applicable, background, objectives, data sources, study eligibility criteria, participants, interventions, study appraisal and synthesis methods, results, limitations, conclusions and implications of key findings, systematic review registration number | 2 |
| INTRODUCTION | | | |
| Rationale | 3 | Describe the rationale for the review in the context of what is already known | 3 |
| Objective | 4 | Provide an explicit statement of questions being addressed with reference to participants, interventions, comparisons, outcomes, and study design (PICOS) | 3-4 |
| METHODS | | | |
| Eligibility criteria | 5 | Specify study characteristics (such as PICOS, length of follow-up) and report characteristics (such as years considered, language, publication status) used as criteria for eligibility, giving rationale | 4-5 |
| Information sources | 6 | Describe all information sources (such as databases with dates of coverage, contact with study authors to identify additional studies) in the search and date last searched | 4-5 |
| Search strategy | 7 | Present full electronic search strategy for at least one database, including any limits used, such that it could be repeated | 4-5 |
| Selection process | 8 | State the process for selecting studies (that is, screening, eligibility, included in systematic review, and, if applicable, included in the meta-analysis) | 4-5 |
| Data collection process | 9 | Describe method of data extraction from reports (such as piloted forms, independently, in duplicate) and any processes for obtaining and confirming data from investigators | 4-5 |
| Data items | 10 | List and define all variables for which data were sought (such as PICOS, funding sources) and any assumptions and simplifications made | 4-5 |
| Study risk of bias assessment | 11 | Specify the methods used to assess risk of bias in the included studies, including details of the tool(s) used, how many reviewers assessed each study and whether they worked independently, and if applicable, details of automation tools used in the process. | 4-5 |
| Effect measures | 12 | Specify for each outcome the effect measure(s) (e.g. risk ratio, mean difference) used in the synthesis or presentation of results. | 4-5 |
| Synthesis methods | 13 | State the principal summary measures (such as risk ratio, difference in means). | 6-7 |
| Reporting bias assessment | 14 | Describe any methods used to assess risk of bias due to missing results in a synthesis (arising from reporting biases) | 6-7 |
| Certainty assessment | 15 | Describe any methods used to assess certainty (or confidence) in the body of evidence for an outcome | 6-7 |
| RESULTS | | | |
| Study section | 16 | Give numbers of studies screened, assessed for eligibility, and included in the review, with reasons for exclusions at each stage, ideally with a flow diagram | Figure 1 |
| Study characteristics | 17 | Cite each included study and present its characteristics. | 7-10 |
| Risk of bias in studies | 18 | Present assessments of risk of bias for each included study | 8-10 |
| Results of individual studies | 19 | For all outcomes, present, for each study: (a) summary statistics for each group (where appropriate) and (b) an effect estimate and its precision (e.g. confidence/credible interval), ideally using structured tables or plots. | 11-13 |
| Results of syntheses | 20 | Present results of each meta-analysis done, including confidence intervals and measures of consistency | 11-13 |
| Reporting biases | 21 | Present assessments of risk of bias due to missing results (arising from reporting biases) for each synthesis assessed | 11-13 |
| Certainty of evidence | 22 | Present assessments of certainty (or confidence) in the body of evidence for each outcome assessed | 11-13 |
| DISCUSSION | | | |
| Discussion | 23 | Summarise the main findings including the strength of evidence for each main outcome; consider their relevance to key groups (such as health care providers, users, and policy makers) | 13 |
| Limitations | 24 | Discuss limitations at study and outcome level (such as risk of bias), and at review level (such as incomplete retrieval of identified research, reporting bias) | 15-16 |
| Conclusion | 25 | Provide a general interpretation of the results in the context of other evidence, and implications for future research | 16 |
| OTHER INFORMATION |  |  |  |
| Support | 26 | Describe sources of financial or non-financial support for the review, and the role of the funders or sponsors in the review. | 17 |
| Competing interests | 27 | Declare any competing interests of review authors | 17 |

Reporting quality assessed with PRISMA checklist.
