# Supplementary material for: Role of physical activity and sedentary behavior in venous thromboembolism: a systematic review and dose-response meta-analysis
Source: Sci Rep. 2024 Sep 27;14:22088. doi: 10.1038/s41598-024-73616-0 (PMC11437044; doi:10.1038/s41598-024-73616-0)
Supplement: Supplementary file 2 — Supplementary Material 2 [file 41598_2024_73616_MOESM2_ESM.docx]

**Supplementary Table 2** | Summary of MET-h/wk dose assignment calculations

| Study | Frequency | Duration | Intensity | Assigned MET h/wk | Additional information |
| --- | --- | --- | --- | --- | --- |
| Yuan, et al^1^ |  | <10 min/day(5min/day) | 4.5METs | 2.625 | COSM study |
|  |  | 10-30min/day(20min/day) |  | 10.5 |  |
|  |  | 30-60min/day(45min/day) |  | 23.625 |  |
|  |  | >60min/day(75min/day) |  | 39.375 |  |
| Yuan, et al^1^ |  | <10 min/day(5min/day) |  | 2.625 | SMC study |
|  |  | 10-30min/day(20min/day) |  | 10.5 |  |
|  |  | 30-60min/day(45min/day) |  | 23.625 |  |
|  |  | >60min/day(75min/day) |  | 39.375 |  |
| Armstrong, et al^2^ | rarely/never (0) | 45min/session | Any activity=4.5MET | 0 |  |
|  | At most once per wk (1) | 45min/session |  | 3.375 |  |
|  | 2–3 times/wk (2.5) | 45min/session |  | 8.4375 |  |
|  | 4–6 times/wk (5) | 45min/session |  | 16.875 |  |
|  | Daily (7) | 45min/session |  | 23.625 |  |
| Lindqvist, et al^3^ | Never (0) | 45min/session |  | 0 |  |
|  | Go for a walk one or more times a week (4) | 45min/session | Walk=3.3MET | 9.9 |  |
|  | Strenuous exercise every week (4) | 45min/session | Strenuous exercise= 8METs | 24 |  |
| [Lutsey, et al](https://pubmed.ncbi.nlm.nih.gov/?size=200&term=Lutsey+PL&cauthor_id=19910349)^4^ | never or rarely to > 4 times per wk (3.5) | Low: 45min/session | LPA=3MET | 7.875 | Physical activity information^5^ |
|  | never or rarely to > 4 times per wk (3.5) | moderate: such as golf, long walks(45min/session) | MPA=4MET | 10.5 |  |
|  | never or rarely to > 4 times per wk (3.5) | Vigorous: swimming, aerobics (45min/session) | VPA=8MET | 21 |  |
| Evensen, et al^6^ | No exercise (0) |  | 0 | 0 |  |
|  |  | Light PA 1-3h/wk(2h/wk) | LPA=3MET | 6 |  |
|  |  | Light PA ≥3h/wk(4h/wk) | LPA=3MET | 12 |  |
|  |  | hard PA 1-3h/wk(2h/wk) | hard PA=8MET | 16 |  |
|  |  | hard PA ≥3h/wk(4h/wk) | hard PA =8MET | 32 |  |
| [Stralen, et al](https://pubmed.ncbi.nlm.nih.gov/?size=200&term=van+Stralen+KJ&cauthor_id=18179500)^7^ | No exercise (0) |  |  | 0 | Physical activity information^8^ |
|  |  | Mild physical activity such as walking (5h/wk) | Mild PA walking  (MET=3.3) | 16.5 |  |
|  |  | Moderate: some physical activity ≥4h/wk (6h/wk) | MVPA (MET=4.5) | 27 |  |
|  |  | high: vigorous physical activity≥3  hours a wk hours a wk, e.g. jogging (4.5h/wk) | VPA (MET=8) | 36 |  |
| [MacDonald, et al](https://pubmed.ncbi.nlm.nih.gov/?size=200&term=MacDonald+CJ&cauthor_id=34419051)^9^ |  |  | < 34.3 METs (17.15) | 17.15 |  |
|  |  |  | 34.3–57.8 METs (46.05) | 46.05 |  |
|  |  |  | > 57.8 METs (69.55) | 69.55 |  |
| Kunutsor, et al^10^ |  |  | 0 MET hours/year | 0 |  |
|  |  |  | 1-200 MET hours/year (1.92MET/wk) | 1.92 |  |
|  |  |  | >200 MET hours/year (4.81MET/wk) | 4.81 |  |
| [Johansson, et al](https://pubmed.ncbi.nlm.nih.gov/?size=200&term=Johansson+M&cauthor_id=30727768)  ^11^ |  | 0 | inactive（MET=0） | 0 |  |
|  |  | <3.5h/week（1.75） | moderately inactive (MET=3) | 5.25 |  |
|  |  | 3.5-7.0h/week（5） | moderately active (MET=4.5) | 22.5 |  |
|  |  | ≥7h/week（8.75） | Active (MET=8) | 70 |  |
| Olson, et al^12^ | none/week (0) | 0 | MPA=4.5MET | 0 |  |
|  | 1-3 times/week (2) | 45min/session | MPA=4.5MET | 6.75 |  |
|  | ≥4 times/week (5) | 45min/session | MPA=4.5MET | 16.875 |  |

Summary of MET-h/wk dose assignment calculations for the studies including in the dose-response meta-analysis.

MET h/wk exposure levels were assigned from descriptions identified within in the individuals studies.

LPA: light physical activity.

MPA: moderate physical activity.

MVPA: moderate to vigorous physical activity.

VPA: vigorous physical activity.

Black font means the frequency, duration or intensity of physical activity in original studies; Green refers to the frequency, duration, intensity or MET were calculated by according to reported.

Red: means the data was assumed Physical activity information.

1. Yuan S, Bruzelius M, Hakansson N, Akesson A, Larsson SC. Lifestyle factors and venous thromboembolism in two cohort studies. *Thromb Res.* 2021;202:119-124.

2. Armstrong ME, Green J, Reeves GK, Beral V, Cairns BJ, Million Women Study C. Frequent physical activity may not reduce vascular disease risk as much as moderate activity: large prospective study of women in the United Kingdom. *Circulation.* 2015;131(8):721-729.

3. Lindqvist PG, Epstein E, Olsson H. The relationship between lifestyle factors and venous thromboembolism among women: a report from the MISS study. *Br J Haematol.* 2009;144(2):234-240.

4. Lutsey PL, Virnig BA, Durham SB, et al. Correlates and consequences of venous thromboembolism: The Iowa Women's Health Study. *Am J Public Health.* 2010;100(8):1506-1513.

5. Kushi LH, Fee RM, Folsom AR, Mink PJ, Anderson KE, Sellers TA. Physical activity and mortality in postmenopausal women. *JAMA.* 1997;277(16):1287-1292.

6. Evensen LH, Isaksen T, Hindberg K, Braekkan SK, Hansen JB. Repeated assessments of physical activity and risk of incident venous thromboembolism. *J Thromb Haemost.* 2018;16(11):2208-2217.

7. van Stralen KJ, Doggen CJ, Lumley T, et al. The relationship between exercise and risk of venous thrombosis in elderly people. *J Am Geriatr Soc.* 2008;56(3):517-522.

8. Brown WJ, Bauman AE. Comparison of estimates of population levels of physical activity using two measures. *Aust N Z J Public Health.* 2000;24(5):520-525.

9. MacDonald CJ, Madika AL, Lajous M, Canonico M, Fournier A, Boutron-Ruault MC. Association between cardiovascular risk-factors and venous thromboembolism in a large longitudinal study of French women. *Thromb J.* 2021;19(1):58.

10. Kunutsor SK, Lakka TA, Kurl S, Makikallio TH, Jae SY, Laukkanen JA. Leisure-time cross-country skiing and the risk of venous thromboembolism: A prospective cohort study. *Eur J Prev Cardiol.* 2020.

11. Johansson M, Johansson L, Wennberg P, Lind M. Physical activity and risk of first-time venous thromboembolism. *Eur J Prev Cardiol.* 2019;26(11):1181-1187.

12. Olson NC, Cushman M, Judd SE, et al. American Heart Association's Life's Simple 7 and risk of venous thromboembolism: the Reasons for Geographic and Racial Differences in Stroke (REGARDS) study. *J Am Heart Assoc.* 2015;4(3):e001494.
